# Syphilis

**Published:** 1878-06

**Authors:** 


### Summary.

*Collaborators:* Dr. H. Gradle, Dr. R. Park, Dr. L. W. Case, Dr. R. Tilley.

### Syphilis.

Abstract of a Series of Four Lectures on Hereditary Syphilis Delivered at the Hospice des Enfants Assistés. By M. Parrot. (*Le Progrès Médical*, Nos. 44 and 47, 1877, and Nos. 1 and 4, 1878.)—Parrot introduces his subject with a brief sketch of the history of the literature of hereditary syphilis; commencing with Gaspard Torella (1498) and Matthiole (1536), who concluded that the disease was induced by the milk of infected nurses, and proceeds to the consideration of abortion due to syphilis.

Menstrual irregularities and suppressions in the subjects of syphilis. are thought by Fournier to be due to the anæmia and cachexia induced by the disease, precisely as other maladies operate to produce the same effect. In this result, however, Parrot believes the specific disorders of the utero-ovarian apparatus play an important part.

Abortion occurs in a little more than one-third of all pregnant women who are syphilitic. The epoch of the abortion depends upon the age of the syphilis in the woman, and, when recent, upon the period of pregnancy when infection occurred. The more complete the term of pregnancy when contamination happens, the fewer are the chances of abortion: at the fifth month, Parrot believes infection rarely interferes with the gestation.

According to Kassowitz, untreated syphilis of the mother for the first three years of the disease, always leads either to abortion or the birth of children which survive but a brief time. Bärensprung is of opinion that it is especially at the 3d, 4th and 5th months that these accidents ensue. Weber, in 1875, had observed

109 pregnant syphilitic women, one-fifth of whom aborted, generally between the 7th and 8th month.

Parrot, after citing various authorities, expresses no opinion as to the relative share of the parents in this premature expulsion of the fœtus, but agrees with all observers in the conclusion that the condition of the product of conception itself is the immediate cause. Babington, Trousseau and Bärensprung believed that the death of the fœtus was the exciting cause; Kassowitz concludes that this is not essential, the greater or less disturbance of its nutrition being sufficient. The lecturer laid no stress upon the anatomico-pathological conditions of the placenta, inasmuch as these are not well understood.

In the matter of treatment, Weber found that 35 women treated by mercurial injection had a normal conclusion of gestation. Of those subjected to mixed treatment (with preponderent employment of potassic iodide) 20 per cent. aborted; of those who simultaneously ingested mercuric bichloride and potassic iodide, 15 per cent., while of those who took potassic iodide only, 36 per cent. aborted.

Exceptionally, the newly-born syphilitic infant bears the evidences of its disease upon its external surface; in such cases death usually supervenes rapidly. As a rule, the syphilitic child at birth appears to be healthy. It has a moderate degree of embonpoint, exhibits a rosy or slightly marbled tint of the skin, its flesh is firm, its cry vigorous, it takes the nipple well, its stools are normal and its urine clear and abundant. This lasts a fortnight, three weeks, or a month, and then the scene changes. A yellowish discharge from the nostrils accumulates around and obstructs their orifice. Suction of the nipple becomes difficult, painful, and accompanied by cries and agitation. The infant commences to waste. Soon the nates, the upper and posterior surfaces of the thighs, and the periphery of the mouth, the nostrils and the chin, become covered with an eruption. At the commissures of the lips, fissures and ulcerated papules form.

The eruption becomes rapidly more abundant and salient. Macules are replaced by red or rosy patches, sometimes of a violet tint, which has been compared to the color of the lean part of a ham, with depressed and greyish center, sometimes scaly or ulcerated, according to its location. Here and there, especially upon the face, brown or reddish crusts appear. The appetite is sensibly diminished ; there are frequent stools and vomiting, the dejections of a greenish color and mixed with mucus. The flesh becomes less firm, the integument loses its tint of health, and has a wrinkled look. When the emaciation has somewhat advanced, by examining with the hand the tissues about the inferior extremity of the arm and the internal face of the leg, it can be determined that the humerus is thickened and the tibia is more voluminous than natural, as though something had been added to the thickness of the bone.

The phenomena which succeed are different, according as the disease assumes a chronic or rapidly fatal phase. In the former case, the eruption both extends to new portions of the integument and becomes more prominent where it had heretofore existed. The buttocks, scrotum, labia and thighs become covered with elevated and indurated patches, resulting in deep and extensive ulcers. From the eyes, the nose, the ears, and the facial lesions, a puriform matter escapes, which concretes into thick irregular crusts, producing a most repulsive aspect of the visage, whose features are also disguised by the swelling of the skin. The eyes become closed, the eyelids glued together, the nostrils obstructed and the lips, which are seamed with deep fissures, bleed on the least contact.

Just before death, a notable change occurs. All the lesions subside and lose color, the redness disappears and the discharge ceases. Only the crusts persist, and even these seem to have lost in volume. At this moment, one who considers the skin alone might conclude that there was an amelioration of the symptoms ; in reality, death is imminent.

In the acute form, the phenomena last described are speedily noted. There is no time for the slow evolution of syphilis ; some complication or intercurrent affection proves fatal, occasionally even before the identity of the specific disease has been established.

Death, however, is not a necessary result. Under favorable conditions of hygiene and treatment, recovery takes place ; macules disappearing first, papules becoming depressed and fading, ulcers healing, often with a permanent scar as the result. The processes of nutrition, temporarily disturbed, resume a normal activity, the flesh becomes firm and the skin assumes a healthy tint. It is the digestive tube which works this marvel, and it is to it, therefore, that the physician should chiefly direct his attention.

Parrot, reviewing the symptoms detailed above, recurs to the subject of the bullous syphilide, commonly called *pemphigus syphiliticus*, as the most precocious of these symptoms. occurring in a large number of cases at birth, and often dating back to the sixth or seventh month of intra-uterine life.

Seated generally upon the palms of the hands and the soles of the feet, it is also found upon adjacent parts, as the dorsal face of the fingers and toes, and the inferior surface of the leg (much more rarely upon distant organs such as the ear). In these latter cases, the eruption is usually tardy of occurence, more discrete and less developed.

In the first few days after birth, the extremities are, as a rule, more deeply congested and colored than other parts of the body. Their hue is of a deep violet shade in the new-born affected with bullous syphilides, and venous red patches may be seen upon them, surrounded by a bright red areola, whose epidermis is speedily raised by the accumulation of liquid beneath, which transforms the lesions into bullæ of variable size. Their diameter may equal from two or three mm. to one and a half ctm. Their development may be rapid; and coalescence occur, forming a compound bulla, whose contour is formed by a series of segments of circles. Some resemble the pustules of variola, others contain a greenish fluid. The smaller ones are made tense by their contents: the larger are often partially filled merely, the roof of the bulla being partially collapsed upon the fluid contents beneath.

Two important points are to be noted: 1st, The bullæ most distant from the seat of election have always less distinctive features than others; they are fewer, smaller, and have less abundant yellow contents. Aborted lesions are to be seen near these, the epidermis being scarcely raised, and without subjacent fluid. 2d. The later the eruption after birth, the less distinctly marked is its type. Hence bullous syphilides, late of occurrence, and seated elsewhere than upon the site of election, may give rise to doubts in diagnosis.

Once fully developed, a portion of the liquid contents may be absorbed and the remainder concrete into a brownish mass ; or the cuticle may burst or become completely detached leaving an ulcer of various extent upon the corium beneath. These ulcers have a red and sanious floor, are not as a rule deep, but are sometimes crateriform and involve all layers of the skin. Generally at this time the subjects of the disease perish.

In the exceptional cases where a cure has been effected (noted by Depaul, Galligo, Stamm, Hertl, Ollivier and Ranvier), the general turgescence subsides, and the crusts fall, exposing an imperfectly formed epidermis, which is renewed after successive desquamations until it acquires sufficient firmness to persist.

Of all the cutaneous syphilides, the bullous appear at the most fixed time, and are also the most precocious. They have however been noted as late as the 7th and the 18th day, and even at the tenth week.

Bullous syphilides appear simultaneously, rarely by successive crops. Intervals of 15 and 19 days have yet been observed, during which time the lesions first to appear have been completely relieved.

There may be co-existence of other syphilides with bullæ, especially when the latter are tardy of appearance. The lesions are not, therefore, as has been taught, uniformly isolated. The minute, very red papules which are rapidly transformed into pustules, and termed "syphilitic ecthyma," are not really such, but constitute one variety of bullous syphilides. "Syphilitic rupia" belongs to the same category. There is really but one disease which requires to be differentiated from that under consideration. It is the pemphigus of the newly-born.

But pemphigus never commences on the palms of the hands and the soles of the feet. If it occur in these situations, it has always first appeared on the neck, axillæ, and upper surface of the thorax, its site of election. In the syphilitic form, the maculæ, which precede the bullæ, the skin from which they are developed, and their surrounding areolæ, are all of a violaceous tint; in non-specific pemphigus the color is a rosy red. The latter, too, are larger and contain at the outset a transparent amber-colored serum, which never becomes purulent, made up eventually of water and protein granules with few epidermic cells and leucocytes. Afterward the fluid is either absorbed or results in a thin impetiginous crust, whose fall exposes a delicate layer of epidermis. In syphilis, the bullæ contain pus, and solid elements predominate in the form of fibrinous granules, pus globules, and whitish flocculi, which are the débris of the mucous layer of the epidermis. Then follows a brownish crust covering an ulcer of the corium. Lastly, syphilis may exist at the moment of birth; pemphigus rarely appears before the fifteenth day, and is frequently observed during the course of the first year.

Is this latter eruption really syphilitic? Parrot thinks it is so unquestionably. The earlier observers so considered it, and also many of those succeeding them. Some have believed it to be due simply to the cachexia engendered by the specific disease. But cachectic pemphigus is quite different in its date of appearance, seat, external phenomena, and histological lesions. As for the opinion that the disease is sometimes produced by syphilis and sometimes by another cause, Parrot dismisses it with a single sentence, and does not believe that when bullæ are the sole manifestation of syphilis, the diagnosis should be doubtful. Observation teaches this truth. In the large number of infants examined by the author, when pemphigus existed upon other regions of the body than the palms of the hands and the soles of the feet, no visceral nor osseous lesions were in any case discovered.

Exceptionally, a bullous eruption occurs in *acquired* syphilis, the "pemphigoid pustular syphilide," of Alibert. Ricord has seen this once on the soles of the feet; Bassereau once also on the palms of the hands. Zeissl in 20 years never saw it. Morgan reports one case, in a woman 26 years of age.

From this fact it will be seen that the bullous syphilide is peculiar to the newly born, and is one of the most characteristic evidences of hereditary disease.

Gummatous Tumor of the Ocular Conjunctiva. Dr. A. M. Berger. (*Acrztl. Intelligenzsbl.* No. 17, 1878).—On Nov.

8, 1877, Mrs. M. H., aged 30, married for two years, consulted the Doctor on account of mydriasis of the left eye, which had occurred quite suddenly. The eye was not irritated; vision and fundus normal, only the pupil was completely dilated and immovable. At the same time a few copper-colored maculæ were noticed on the forehead, and although the patient denied any possibility of infection the suspicious maculæ suggested a constitutional treatment. Under the free use of iodide of potassium, and the occasional application of eserine, the mydriasis disappeared after three weeks.

But in January last, the lady returned with a squamous syphilide over the forehead, temples and eyelids; adenopathy, and a violent acute iritis. The peculiar feature of the case was a grayish white nodule at the inner margin of the cornea; it was 3 mm. broad and 2 mm. high in its center, gradually sloping off toward the cornea and sclerotic. The conjunctiva passed over its smooth surface. After one week of mercurial treatment this subconjunctival nodule began to get smaller, while, with an aggravation of the iritis, two small brownish condylomata appeared on the inflamed iris. On February 15, however, condylomata, gumma, and cutaneous syphilide had disappeared. The situation of the gumma was indicated by a yellowish discoloration of the sclerotic.

